# Opium

**Published:** 1871-08

**Authors:** R. Rother


*Opium.* By R. Rother.

The great importance of opium as a medicinal agent requires, and the peculiarity of its constitution admits, a very definite adjustment of its power. It has long been the effort of leading pharmaceutists to popularize a system for definitely standardizing the activity of crude drugs or their preparations. But only a very limited number of these substances have thus far yielded desirable results by the practically applicable methods of analysis at present known.

It is exceedingly fortunate, however, that opium—one of the most valuable in the materia medica list—can be so easily and accurately assayed. The standard of quality is based upon the quantity of its chief constituent, morphia, and the method of isolating it for this purpose is founded upon the original process of Sertuerner, by whom "the alkaloid" was discovered.

Among the numerous alkaloids and other principles of opium, are several besides morphia of acknowledged power, as opianina and papaverina, for example, but these are found only in minute proportion. There is another at least of medium power, but much inferior to morphia both in effect and quantity, called codeia, and another occurring copiously, but medicinally inert, named narceina, and one entirely destitute of narcotic properties, performing the function of a powerful tonic merely, and strangely denominated narcotina, in quantity exceeding all the others but morphia. Together with these a considerable quantity of undetermined or unknown compounds of a more or less active character, complete the list of ingredients to which opium owes its activity, and for all, taken as a whole, the gauge is the morphia strength.

The serious nervous disturbance following the usual effect of opium on the system, is attributed to an exceedingly subtle and as yet undetermined substance. But it has been found that ether will thoroughly remove this noxious and objectionable body. In consequence, a number of opium preparations, proprietary and otherwise, are in general use, all differing from ordinary opium compounds, by the reason that the preliminary action of ether was resorted to. The noxious substance, however, seems to be but sparingly soluble in water; so, then, a preparation made with an aqueous extract of opium, may possess an approximate identity to one made of opium purified by ether.

The elegant and unsurpassable deodorized tincture of opium of the Pharmacopœia, is as yet comparatively unknown, but its virtue is only apparently eclipsed by similar yet secret preparations, whose only merit over it is the advantage derived from a priority of introduction into general use through the customary system of advertising, and probably, also, a uniformity in morphia strength, determined by assay.

Although it is an officinal requirement that all opium entering into the compounds of the Pharmacopœia should contain seven per cent. of morphia, the direction is rarely observed, and therefore an inferior opium employed otherwise according to the Pharmacopœia, will necessarily yield but a worthless or very weak preparation. However, now it is generally agreed that when opium is directed officinally, as in the preparation of Tincture of Opium, it should contain ten per cent. of morphia, or four grains of the pure alkaloid in the fluid ounce of the tincture.

Comparatively simple as the process of isolating the morphia for the purpose of assay may be, it still requires especial precaution and considerable experience to obtain strictly reliable results. In view of this fact we have numerous methods, each with its particular advantages, proposed to attain the desired end. But in most cases, and especially in doubtful ones, two separate assays are usually performed, and if possible by different yet equally accurate methods. To obviate this inconvenience, and insure a perfectly conclusive result at once, the number of these processes still accumulates.

The most prominent methods of assay are as follows:

The process of Merck is to boil half an ounce of opium first with eight ounces, then with four ounces of proof spirit, filtered, and the filtrates evaporated to dryness after the addition of two drachms of disodic carbonate. The brown residue is introduced into a tall cylindrical glass vessel and softened with water, the colored supernatant liquid poured off, the residue again washed with water and then brought in contact, for one hour, with cold alcohol, sp. gr. .85. The mixture is now poured upon a filter, the residue washed with alcohol, and then dissolved in half an ounce of distilled vinegar previously mixed with its bulk of water, the morphia again precipitated from the filtrate by the addition of a slight excess of ammonia, and after twenty-four hours the precipitate is poured into a filter, dried and weighed.

Duflos exhausts opium with cold water, adds one-eighth of its weight of monopotassic carbonate, and sets the mixture aside for twelve hours. The liquid is then filtered from the precipitated narcotina, and boiled until the effervescence of carbon dioxide has ceased. After twenty-four hours the morphia has all precipitated as a crystalline powder, and in order to free it entirely from adhering narcotina, it is again dissolved in very dilute sulphuric acid, the solution mixed with alcohol, until the whole liquid weighs nine-fourths as much as the opium employed, and the morphia reprecipitated with a slight excess of ammonia.

According to Mohr, the opium is three times successively treated with water, the liquids united, evaporated, filtered and poured into boiling milk of lime, containing one-sixth to one-quarter as much calcium hydrate as the weight of the opium used. After boiling a few minutes the mixture is poured on a linen strainer, the precipitate washed with boiling water, and finally pressed. The strained wine yellow liquid is now reduced by evaporation to twice the weight of the opium, and filtered if necessary. This is now heated to boiling and the morphia precipitated by addition of ammonium chloride equal to one-sixteenth of the opium used. By solution in dilute chlorhydric acid and treatment with animal charcoal, the morphia can be further purified.

By a fourth method, the aqueous infusion of opium is treated with plumbic acetate as long as a precipitate occurs; this is collected on a filter, and washed with water until the filtrate is not perceptibly bitter. The excess of lead is now precipitated from the filtrate with sulphydric acid; the lead sulphide left in contact with the liquid for some time, then filtered, and the wine yellow filtrate cautiously treated with ammonia. A resinous precipitate containing narcotina is thereby separated, which adheres tenaciously to the walls of the glass. The liquid is now poured off, and the morphia precipitated by a further addition of ammonia.

Instead of using water the opium can also be exhausted with dilute acetic acid, the liquid repeatedly evaporated, after adding fresh portions of water until the excess of acid is expelled, and the narcotina, together with extractive, has become insoluble. The morphia is then precipitated with ammonia, washed with water and alcohol, and finally crystallized from absolute or ninety per cent. alcohol.

According to Thiboumery, opium is three times successively treated with cold water, and the infusions evaporated to a syrupy consistence. The extract is mixed with water, poured on a filter, and the residue washed with water until decolorized. The filtrate is now boiled and precipitated with ammonia; after cooling, the precipitate is filtered off, washed first with cold water then with alcohol, sp. gr. .94, until decolorized, and dissolved in boiling alcohol, sp. gr. .84, with the addition of animal charcoal, filtered, and the morphia crystallized from the solution.

Guilliermond rubs the opium, first broken into small fragments, three times with three times its weight of alcohol, 71 per cent. and filters into a wide mouthed bottle containing ammonia equal to one-quarter the opium used. After twelve hours, rather large colored crystals of morphia separate, together with small shining crystals of narcotina. The crystals are then poured on a linen strainer, washed with water, dried, and then thrown into water; the morphia sinks to the bottom, whilst the narcotina remains suspended and can be poured off.

By the method of Fordos, the opium, cut in slices, is first macerated with four times its weight of water, for twenty-four hours. The mixture is then thoroughly rubbed up in a mortar and poured on a filter; when the liquid has flowed off, a second and then a third portion of water, equal in weight to the opium, is poured on.

One-third of the mixed liquids is then precipitated with ammonia, to determine the requisite amount for the remaining two-thirds. To the remaining liquid an equal volume of alcohol, 85 per cent., is added, and twice the quantity of ammonia first used. The mixture is now agitated and transferred to a bottle, well stopped, and allowed to stand for two or three days. The deposited crystals are now poured on a filter, washed with weak alcohol, about 40 or 50 per cent., and then treated with ether, after this with chloroform, and again with ether; after drying, the crystals are weighed as pure morphia.

By Staples' process (the officinal process), opium is first macerated with five times its weight of water for forty-eight hours, then exhausted with cold water, the infusion evaporated to about eight times the weight of the opium, and about an equal volume of officinal alcohol added; to this an excess of ammonia, previously mixed with alcohol, is added in two portions, after an interval of twenty-four hours; at the end of twenty-four hours, the deposited morphia is first washed with a little diluted alcohol, then with water, dried and weighed.

From these various processes, we observed that the best method for exhausting the opium of its morphia is the treatment with cold water, preceded by a maceration; it is also seen that ammonia is the final precipitant in every case, and, with the exception of the third and fourth methods, alcohol is used in every instance to decolorize the morphia. In nearly every process the adhering narcotina is separated by again dissolving the impure morphia in some dilute acid, and precipitating it a second time. But in the method of Fordos, ether and chloroform are used for this purpose.

Now, to understand the relative value of all these processes, it must be known that morphia is insoluble in alkaline carbonates, quite soluble in ammonia (one grain dissolving in about 100 of the ordinary 18 per cent. ammonia), soluble in 1,000 parts of cold water, and in 500 when hot; it is also soluble in about 100 parts of strong alcohol, which solubility gradually approaches that of water as the alcohol is weaker; it is insoluble in ether, but soluble in 60 parts of chloroform. One grain of pure morphia will also dissolve, after a few moments shaking, in a mixture of five fluid drachms of strong alcohol, four fluid drachms of water, and one fluid drachm of chloroform. But the same quantity of morphia is scarcely if at all diminished by a mixture of four fluid drachms each of strong alcohol, water and ether. The writer believes that the exceeding solubility of morphia in chloroform may sometimes be due to acidity of the latter, and, therefore, chloroform should never be used for purifying the morphia in opium assays. Ammonia, although used in all the processes, is objectionable because an excess is required first to separate the morphia completely; and as it is difficult to ascertain the exact amount in each case, an immoderate excess may again dissolve some of the morphia or prevent its precipitation. For this reason, a fixed alkaline carbonate is greatly preferable as a precipitant, and its employment for this purpose really constitutes the second stage in the methods of Merck and Duflos. Aside from the use of alkaline carbonate, these two processes—but especially the original and ingenious one of Duflos—also furnish the ground work for all the others, with the exception of the second stages of Mohr's and the fourth method above given. The methods of Guilliermond, Fordos and Staples, are also very objectionable, firstly, that the morphia is precipitated from solutions that are too dilute, and, secondly, that these solutions are alcoholic; because, if even two and a half fluid ounces of water will retain one grain of morphia in solution, the same volume of alcoholic liquid will dissolve much more. Moreover, morphia will remain in such a solution more abundantly than if it were precipitated as a crystaline powder from a concentrated aqueous solution, and then treated with the same volume of these solvents.

If in an alcoholic solution ammonia is used as a precipitant, it is utterly impossible to know when the proper quantity has been added, unless determined by a preliminary trial according to Fordos. However, the writer has found the case to be quite different with a concentrated aqueous solution. If to such a solution ammonia, moderately dilute, be added drop by drop, with constant stirring, the gelatinous precipitate at first produced immediately contracts and rapidly subsides the moment a sufficiency or slight excess of ammonia has been added. The writer thinks this to be an exceedingly practical and important indication in the use of ammonia as a precipitant. The same observation was made with disodic carbonate, only the precipitation is less rapid at first than with ammonia.

Morphia exists in opium combined with sulphuric and meconic acid, but often entirely with the former when the latter is absent. The meconic acid is tribasic, and the alkaloid, or any other monad base, can form with it either monobasic, dibasic or tribasic meconate. The trimetallic salts are usually the most soluble, and the monometallic, or so-called acid salts, the least. Morphia originally exists in the opium when in combination with meconic acid, always as the tribasic or normal salt, owing to the constant deficiency of meconic acid. When morphia is precipitated by ammonia or disodic carbonate from its native union with meconic acid, the reaction may take place, as is shown in the following equations:

$$C_7H\ (C_{17}\ H_{19}\ NO_3)\ _3O_7 + 3\ (CO_3\ Na_2) + 3\ (H_2O) = 3\ (C_{17}\ H_{19}\ NO_3 . H_2O) + C_7H\ Na_3\ O_7 + 3\ (CO_3\ NaH)$$

$$C_7H\ (C_{17}\ H_{19}\ NO_3)\ _3O_7 + 2\ (CO_3\ Na_2) + 3\ (H_2O) = 3\ (C_{17}\ H_{19}\ NO_3 . H_2O) + C_7\ H_2\ Na_2\ O_7 + 2\ (CO_3\ NaH)$$

$C_7H\,(C_{17}\,H_{19}\,NO_3)\,_3O_7+CO_3\,Na_2+3\,(H_2O)=3\,(C_{17}\,H_{19}NO_3.\,H_2O)$

$+C_7\,H_3\,Na\,O_7+CO_3\,NaH$

The writer, after numerous trials of these various methods, has devised a process which, for accuracy, speed and simplicity, is equal to any and superior to most, and therefore recommends itself for the use of pharmaceutists generally.

This process is a modification of the methods of Merck, Duflos and Fordos. The opium, if moist or in powder, is first subjected to maceration, then transferred to a filter and treated with cold water until thoroughly extracted, the filtrate evaporated to a small bulk and treated (without filtration, because the sediment of narcotina, if any, is effectually removed in the subsequent part of the process) with a concentrated solution of disodic carbonate. This precipitates, besides morphia and the usual amount of coloring matter and narcotina, nothing that water, alcohol and ether cannot dissolve. Opium contains no soluble mineral matter that could possibly enter the aqueous solution, and then be precipitated as an insoluble carbonate, unless such were fraudulently added, which is not known to be the case. However, it is worth while here to state that if glycerin were added to keep the opium moist, all the present methods of assay would be entirely unavailing, as the glycerin would retain much of the morphia in solution, despite ammonia or disodic carbonate. After six to twelve hours, the precipitate is poured on a balanced filter, first washed with a little water, then with a mixture of equal measures of strong alcohol, water and ether, until decolorized, dried and weighed. The first objection that might be raised against this process is, that the morphia is at once precipitated from the concentrated liquid obtained by evaporating the aqueous infusion. But the writer has found that it is utterly useless to filter this liquid, because only loss of time and material is incurred, and nothing whatever gained. The greatest amount of sediment in such a solution—resulting from 200 grains of opium—that the writer ever noticed, weighed only three grains, and was completely soluble in diluted alcohol. The second objection might be, that the morphia obtained by precipitation with alkaline carbonate can be contaminated with earthy carbonates, but the precipitate is perfectly soluble in caustic alkali.

The peculiar advantages of this process are, firstly, that the morphia solution is never removed from the vessel in which it is concentrated.

Secondly, that the precipitant can be used even in large excess without danger of disolving the morphia.

Thirdly, that when the morphia is precipitated from a concentrated aqueous solution, it is less colored and much more easily and thoroughly decolorized subsequently than when precipitated from a concentrated alcoholic solution.

Fourthly, that with an aqueous solution no extra precautions need be taken to prevent inordinate evaporation, as is the case with an alcoholic liquid; the dish requires simply to be covered and set aside, to give the morphia time for complete separation.

Fifthly, that the morphia is poured on a tared filter, from which it is not removed again until washed, dried and weighed.

Sixthly, that the morphia is not decolorized with weak alcohol in one operation, and freed from adhering narcotina by means of ether in another; but that this operation is performed in one process more expeditiously and thoroughly by the use of a mixture of alcohol, ether and water, with which the morphia is thoroughly mixed by means of a small glass rod after each new addition of the mixture.

The necessary apparatus, when reduced to a practical minimum, is summed up in an ordinary druggists' prescription scale that will weigh accurately to the fourth of a grain; a four-ounce porcelain capsule, a small earthernware pestle, a very small and blunt edged glass rod, a 5-inch ordinary filter, two 4-inch filters, which, after being dried, must be exactly equal in weight, a small glass funnel with a slant height of two and a half to three inches, an ordinary six or eight ounce wide mouthed bottle, a small, flexible steel spatula, a piece of sheet-iron covered with a thin layer of sand for a sand-bath, and any vessel of convenient size and shape containing hot water, to serve as a water-bath.

The assay is now performed as follows:

Two hundred grains of opium—whether in powder or moist lump—is always taken, for the object of greater accuracy. If it be in the latter form, it must first be broken into small fragments before weighing. It is then placed in a porcelain capsule of convenient size, and two fluid ounces of cold water poured on; after a maceration of several hours, the mass is stirred up with a small pestle, and this operation frequently repeated during a maceration of six or eight hours, or until the fragments have become perfectly disintegrated; one or two fluid ounces of cold water are now added, stirred up, and the magma poured into a five or six-inch filter, previously adjusted in a funnel and moistened with water; when the liquid, which is collected in a six or eight-ounce wide mouthed bottle, has flowed off, more water is poured unto the opium, some of it having first been used to wash the capsule and pestle. Immediately after each addition, the mass on the filter is carefully stirred up with a small glass rod, and this is several times repeated in the beginning, but latterly the residue is left at rest, and water continuously poured on until twelve fluid ounces of filtrate has accumulated. This liquid is now poured into the capsule again, in portions of two or three fluid ounces—as fast as the filtrate collects—placed on a sand-bath moderately heated, and evaporated to about one and a half fluid ounces. After cooling, forty to sixty grains of disodic carbonate, previously dissolved in two fluid drachms of water, is added, stirred with a glass rod, the mixture covered up and set aside for six to twelve hours to give the morphia time for complete separation. Two four-inch filters, well dried, are now exactly balanced, one of them laid aside and the other placed in the funnel and moistened with water. The liquid with the precipitated morphia is now stirred with a spatula and poured into the filter. By means of the spatula the precipitate adhering to the dish is perfectly removed, and washed into the filter with a portion of the filtrate already collected; the dish is then further washed with small portions of distilled water, and the washings also rinsed into the filter. The precipitate, after being washed with more water, using about four to six fluid drachms in all, is then treated with a mixture of five or six fluid drachms each of strong alcohol, water and ether, until the filtrate is colorless, or nearly so; the above quantity is about sufficient for the purpose. The mixture also must have perfectly cooled before it is used. A portion is first poured on to the precipitate and carefully stirred with a glass rod, and the process repeated after each addition of the liquid, until finally the precipitate contracts, assumes a granular appearance and falls to the bottom of the filter. The stirring up of the precipitate is important in this part of the process, as through it a very effectual washing of the morphia is secured with the least possible quantity of menstruum. The morphia can also be decolorized, first with a mixture of one fluid ounce each of strong alcohol and water, and the narcotina then removed by washing with ether. But then it requires much more of the latter (about two fluid ounces), and the morphia must always be digested a short time with each new portion added. The filter containing the morphia is now detached from the funnel, laid on several thicknesses of unsized paper for a short time to remove most of the adhering liquid by absorption, and then dried, either by exposure in a warm place, but best on a water bath by being placed on a sheet of filtering paper in a porcelain dish, floated on hot water. The empty and also dry filter is then laid on one side of the balance, and the one holding the precipitate in the other; the weight of the morphia thus obtained is divided by 2, and the result indicates the percentage in grains. The morphia is in the form of a grayish-white granular powder.

When making preparations of opium—as laudanum, for instance,—it is necessary to exhaust the opium first with the menstruum; then using a quantity of the liquid representing 200 grains of opium, evaporating off the alcohol, diluting the residue with water, filtering to separate the resinous matter dissolved by the alcohol, evaporating the filtrate to a small bulk, and determine the morphia as above directed. From this the whole amount of morphia in the concentrated liquid is calculated, and this diluted so that every fluid ounce of the diluted liquid, or in other words laudanum, shall contain four grains of pure morphia in the fluid ounce. If the opium should be deficient in morphia, and the first liquid obtained have less than four grains in the fluid ounce, then the requisite quantity of an assayed opium must be added to furnish the necessary amount of morphia.

In adjusting the strength of deodorized tincture of opium, a quantity of the aqueous infusion, before evaporation, corresponding to 200 grains of opium, is treated precisely according to the above method of assay from the beginning. The writer believes that although the ether removes the odorous substance, but very little of the narcotina is removed, and consequently that would contaminate the morphia unless this were subjected to the whole routine of the assaying process.

The writer has observed that when an aqueous solution of opium is treated with ammonia or disodic carbonate, the characteristic fruity odor of deodorized tincture of opium is almost instantly developed; the same occurs, but more slowly, when an alcoholic solution of opium is first largely diluted with water and then subjected to the same agency. This is also effected in an ordinary alcoholic solution, but the deodorization progresses very slowly. These indications would second the plausible view that the fine aroma and the so-called narcotic odor of opium are due to two distinct substances, existing originally side by side in the opium, but that the extreme delicacy of the former is completely concealed by the powerful predominance of the latter. It is almost certain that the substance possessing the narcotic odor undergoes an entire change in contact with the alkaki, because it cannot be reproduced by the subsequent addition of an acid, which would probably be the case were it an acid body that had combined with the alkaline base. It is highly probable, then, that the noxious body removed by the ether is the substance destroyed by the alkali. If this were true, the deodorized tincture of opium could be much more economically and expeditiously prepared, by adding ammonia or disodic carbonate, and then restoring the disturbed equilibrium again by neutralization with sulphuric acid.

The magnificent and useful preparation called "Liquor Opii Compositus," invented by Dr. Squibb, is much used in some localities. But the process of Dr. Squibb is too scientific for general use. About six months ago the writer had occasion to prepare some, and in order to render the process practicable was compelled to make some alterations. This article at the present time is as beautifully clear and fragrant as when first prepared.

Dr. Squibb's preparation is of the laudanum strength in morphia—that is, one fluid ounce contains four grains of the pure alkaloid, equal to five grains of the sulphate, and thirty minims of it are composed as follows:

| | |
|---|---|
| An assayed so-called depurated solution of opium | 14 minims. |
| Strong Alcohol | 13 " |
| Chloroform | 1 minim. |
| Acetic Ether | 2 minims. |

This gives 16 minims of chloroform and 32 minims of acetic ether in the fluid ounce. But the writer was called upon to furnish it at once, and seeing that it was practically possible to comply, prepared it extemporaneously as follows: Having at hand deodorized tincture of opium, acetic ether, and Dr. Murdock's solution of chloroform in glycerin, containing 17 minims of chloroform in the fluid drachm.

For one fluid ounce of Liquor Opii Compositus, approximately, Take of

| | |
|---|---|
| Deodorized tincture of opium | 7 fluid drachms. |
| Dr. Murdock's chloroformic solution | 1 fluid drachm. |
| Acetic ether | 32 minims. |
| Mix. | |

The writer believes with Dr. Squibb that in this preparation the alcohol should be a minimum. Therefore, a preparation having the alcoholic strength of deodorized tincture of opium, or even weaker, is much better. This is secured by the use of Dr. Murdock's solution of chloroform in glycerin, and the process given by him is as follows:

Place 3 fluid ounces of glycerin in a conveniently sized mortar, and rub up with it by gradual addition one fluid ounce of chloroform; introduce this mixture into a bottle to let a little undissolved chloroform subside, and then, after separating it, rub this up with another fluid ounce of glycerin, and mix the whole together. This is a permanent solution, and is miscible with water in all proportions.

By this means an accurate formula for Liquor Opii Compositus would be as follows, for 2 pints: Take of the assayed concentrated solution of opium, purified by ether according to the Pharmacopœia, a volume corresponding to 128 grains of morphia.

| | |
|---|---|
| Strong alcohol | 7 fluid ounces. |
| Dr. Murdock's solution of chloroform | 4 " " |
| Active ether | $17\frac{1}{15}$ fl. drachms. |
| Water sufficient. Mix. | |

—*The Chicago Pharmacist.*

